# Inflammatory Role of Toll-Like Receptors in Human and Murine Adipose Tissue

**DOI:** 10.1155/2010/823486

**Published:** 2010-03-22

**Authors:** Odile Poulain-Godefroy, Olivier Le Bacquer, Pauline Plancq, Cécile Lecœur, François Pattou, Gema Frühbeck, Philippe Froguel

**Affiliations:** ^1^CNRS 8199-Institute of Biology, Pasteur Institute, 59019 Lille, France; ^2^INSERM U859, IFR114 IMPRT, Faculté de Médecine, Pôle Recherche, 59045 Lille, France; ^3^Department of Endocrinology, Clínica Universitaria de Navarra, Universitaria de Navarra, Univ. Navarra, and CIBER Fisiopatología de la Obesidad y Nutrición, Instituto de Salud Carlos III, 31008 Pamplona, Spain; ^4^Genomic Medicine, Imperial College London, Hammersmith Hospital, Du Cane Road, London W12 0NN, UK

## Abstract

It was recently demonstrated that TLR4 activation via dietary lipids triggers inflammatory pathway and alters insulin responsiveness in the fat tissue during obesity. Here, we question whether other TLR family members could participate in the TLR-mediated inflammatory processes occurring in the obese adipose tissue. We thus studied the expression of TLR1, TLR2, TLR4, and TLR6 in adipose tissue. These receptors are expressed in omental and subcutaneous human fat tissue, the expression being higher in the omental tissue, independently of the metabolic status of the subject. We demonstrated a correlation of TLRs expression within and between each depot suggesting a coregulation. Murine 3T3-L1 preadipocyte cells stimulated with Pam3CSK4 induced the expression of some proinflammatory markers. Therefore, beside TLR4, other toll-like receptors are differentially expressed in human fat tissue, and functional in an adipocyte cell line, suggesting that they might participate omental adipose tissue-related inflammation that occurs in obesity.

## 1. Introduction

Toll-like receptors (TLRs) are transmembrane receptors initiating a range of host defense mechanisms in response to microbial products [1, 2]. Activation of the TLRs leads to activation of intracellular signaling pathways which results in the production of inflammatory cytokines, or chemokines, inducing the development of antigen-specific adaptive immunity. The TLR family contains 10 members in humans (*TLR1*-10). These receptors recognize pathogen-associated molecular patterns as well as host-derived ligands released by various cell types during immune responses. This leads to signaling events resulting in acute host responses necessary to kill the pathogens [3]. This activation is beneficial for the host but can become deleterious if resulting in chronic inflammation [4]. 

Beside their role in innate and adaptive immune responses, TLRs have been recently described to regulate energy metabolism, mostly through acting on adipose tissue. In particular, it was demonstrated that *TLR4*, the receptor for recognition of gram-negative bacterial cell wall components, was able to sense free fatty acids and to induce insulin resistance in adipose tissue [5]. *TLR2*, as *TLR4*, has been shown to be activated by saturated free fatty acids [6] and is implied in bacterial lipoprotein recognition [7]. Unlike other TLRs, which are functionally active as homodimers, *TLR2* can form heterodimers with *TLR1* or *TLR6* that will recognize distinct molecular patterns of lipopeptides and can discriminate between tri- and diacylated lipopeptides [8, 9]. Our purpose is to demonstrate that beside *TLR4*, *TLR2* and its partners *TLR1* and *TLR6* are potentially susceptible to play also a role in adipose tissue inflammation. 

Epidemiological studies have clearly demonstrated a relationship between intraabdominal fat depots and metabolic abnormalities related to obesity [10, 11]. In this regard, subcutaneous and omental adipose tissues display different metabolic features [12, 13] such as differences in lipolysis or adipokine secretion. Physiologically, TLRs activation in fat cells induces cytokine secretion which triggers further inflammation. Since omental tissue is related to a higher degree of inflammation [14], a high expression of TLRs in this tissue may correspond to an implication of these receptors in obesity-related inflammation. 

We analyzed, *TLR1*, *TLR2*, *TLR6,* and *TLR4* expression in paired human adipose omental and subcutaneous samples from subjects with different glycaemic status.

We previously demonstrated that inflammation correlates with a decrease of lipogenesis [14] and that *TLR4* stimulation interferes with adipocyte differentiation [15]. In order to determine whether *TLR2* activation can be also implied in proinflammatory stimulation or interact with adipogenesis, we studied the impact of Pam3CSK4 (a *TLR1/TLR2* agonist) exposure on 3T3-L1 preadipocyte cell line.

## 2. Subjects, Materials, and Methods

### 2.1. Reagents

Insulin, dexamethasone and, isobutyl-1-methylxanthine were purchased from Sigma Chemical Co. (St Louis, MO, USA). The synthetic bacterial lipoprotein N-Palmitoyl-S-[2,3-bis(palmitoyloxy)-(2RS)-propyl]-[R]-cysteinyl-[S]-seryl-[S]-lysyl-[S]-lysyl-[S]-lysyl-[S]-lysine (Pam3CSK4) was from InvivoGen (San Diego, CA, USA). Tetramethylbenzidine (TMB) was from BD Biosciences (Franklin Lakes, NJ, USA).

### 2.2. Subjects

Forty-one Caucasian female subjects (seven lean volunteers and thirty-four obese patients) attending either the “Département de Chirurgie Générale et Endocrinienne, CHRU de Lille” or the Department of Endocrinology of the Clínica Universitaria de Navarra were enrolled in the study. The subjects were classified as normal weight or obese according the to WHO criteria. Subjects were further classified into 3 groups according to the recently established diagnostic thresholds (based on an Oral Glucose Tolerance test, OGTT) for diabetes and lesser degrees of impaired glucose regulation (normoglycaemia: fasting plasma glucose concentration (FPG) <100 mg/dL and 2-h PG <140 mg/dL after OGTT; glucose intolerant: FPG >100 mg/dL and <125 mg/dL or 2-h PG between 140 and 199 mg/dL after OGTT; type 2 diabetes mellitus: FPG ≥126 mg/dL or 2-h PG ≥200 mg/dL after OGTT; Table 1). The lean group included patients undergoing surgery due to benign diseases, such as cholecystectomy, while the 34 obese patients strictly met the criteria for bariatric surgery. In both groups of patients surgery was performed by the minimally invasive laparoscopic approach. Informed consent was obtained from all subjects and the experimental design was approved by the Hospitals' Ethical Committees responsible for research. All patients underwent a preoperative evaluation including medical history and physical examination. Biopsies were obtained from both subcutaneous and omental adipose tissues of lean and obese volunteers. Fat samples were immediately frozen in liquid nitrogen and stored at −85°C. 

### 2.3. Cell Culture and Induction of Adipocyte Differentiation

3T3-L1 preadipocytes were maintained and cultured in DMEM (Gibco, Paisley, Scotland, UK) containing 10% (vol/vol) fetal calf serum (Gibco). 3T3-L1 cells were differentiated into adipocytes as previously described [16]. Briefly, 2-day postconfluent 3T3-L1 preadipocytes (designated day 0) were fed DMEM containing 10% FCS, 10 *μ*g/mL insulin, 1 *μ*M dexamethasone, and 0.5 mM 3-isobutyl-1-methylxanthine for 2 days. Cells were then fed DMEM supplemented with 10% FCS and 5 *μ*g/mL insulin until day 10.

### 2.4. Evaluation of Gene Expression Levels by Quantitative Real-Time RT-PCR and Quantification of Protein Secretion by ELISA

Human samples were homogenized using an ULTRA-TURRAX T25 basic equipment (IKA Werke GmbH, Staufen, Germany). Total RNA was extracted from 3T3-L1 cells or from human adipose samples using RNeasy Lipid kit (Qiagen, Courtaboeuf, France). One microgram of total RNA was transcribed into cDNA using cDNA Archive Kit (Applied Biosystems, Foster City, CA, USA). Each cDNA sample was analyzed for gene expression by quantitative real-time PCR (qPCR) using the fluorescent TaqMan 5′-nuclease assay on an Applied Biosystems 7900HT sequence detection system. The TaqMan real-time PCR was performed using 2× TaqMan Master Mix and 20× premade TaqMan gene expression assays (Applied Biosystems). Analysis was performed with ABI 7900HT SDS 2.2 Software. For 3T3-L1 samples, the mRNAs levels were normalized to that of acidic ribosomal phosphoprotein (*36B4*), a gene whose expression is unaffected by adipogenesis [17]. For human samples, glyceraldehyde-3-phosphate dehydrogenase (*GAPDH*) was used as reference gene, since it was previously described to exhibit a low coefficient of variation and no significant differences in mRNA levels between samples of the different phenotypical groups [14]. The data are given as the ratio of the target gene mRNA to that of *GAPDH* or *36B4* mRNA level. 

Concentrations of cytokines and chemokines were measured by DuoSet ELISA Development System according to manufacturer instructions (R&D Systems, Abingdon, UK). To further confirm the specific *TLR2* activation and to exclude potential endotoxin contamination of Pam3CSK4 agonist leading to *TLR4* activation, we used a monoclonal anti-*TLR2* antibody to inhibit its biological activity (clone T2.5 from Hycult Biotechnologies, Uden, The Netherlands, IgG1 isotype) [18, 19]. The negative control was performed using another IgG1 monoclonal antibody (15H6, Interchim, Montluçon, France).

### 2.5. Statistical Analysis

Statistical analysis was performed with the SPSS software package (14.0.2, Chicago, IL). According to sample size, the test on ranks was performed and two-tailed exact *P*-values are given. The mRNA levels between lean and obese patients or in cell culture studies were analyzed by U Mann-Whitney's test. Comparisons of mRNA levels between subcutaneous and omental adipose tissues were performed using the paired Wilcoxon test. Correlations between continuous variables were determined using the nonparametric Spearman's rank correlation. The threshold of significance was set at *P* < .05. 

## 3. Results

### 3.1. *TLR1*, *TLR2*, *TLR4*, and *TLR6* Human Adipose Tissue Expression

**Figure 1:**
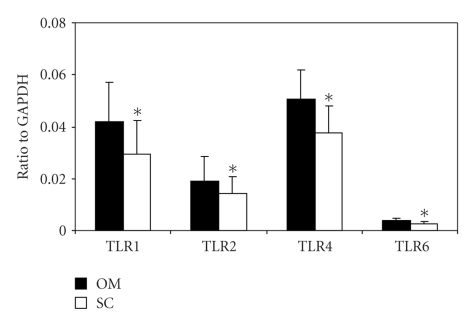
*TLR1*, *TLR2, TLR4*, and *TLR6* mRNA expression in adipose tissues according to localization (OM = omental, SC = subcutaneous;) in human subjects. The data are presented as the ratio of the levels of the target gene mRNA to that of *GAPDH* mRNA. The nonparametric paired Wilcoxon test was performed between subcutaneous and omental values for each gene and demonstrated a significant difference between omental and subcutaneous expression.

We first globally analyzed *TLR1* and *TLR2* mRNA expression in omental and subcutaneous adipose tissue of 34 morbidly obese (BMI = 46.4 ± 5.6 kg/m^2^) and 7 lean subjects (BMI = 21.4 ± 2.4 kg/m^2^) by qPCR. We observed an increased expression of TLRs in omental fat compared to subcutaneous adipose tissue (1.42-, 1.35-, 1.35-, and 1.40-fold increase for *TLR1*, *TLR2*, *TLR4*, and *TLR6*, resp.; Figure 1). The nonparametric paired Wilcoxon test was performed between subcutaneous and omental values for each gene and demonstrated a significant difference. *P*-values are, respectively, *TLR1*: 1,03 10^−6^; *TLR2*: 3.84 10^−4^; *TLR4*: 1,29 10^−6^; *TLR6*: 2.30 10^−6^.

**Figure 2:**
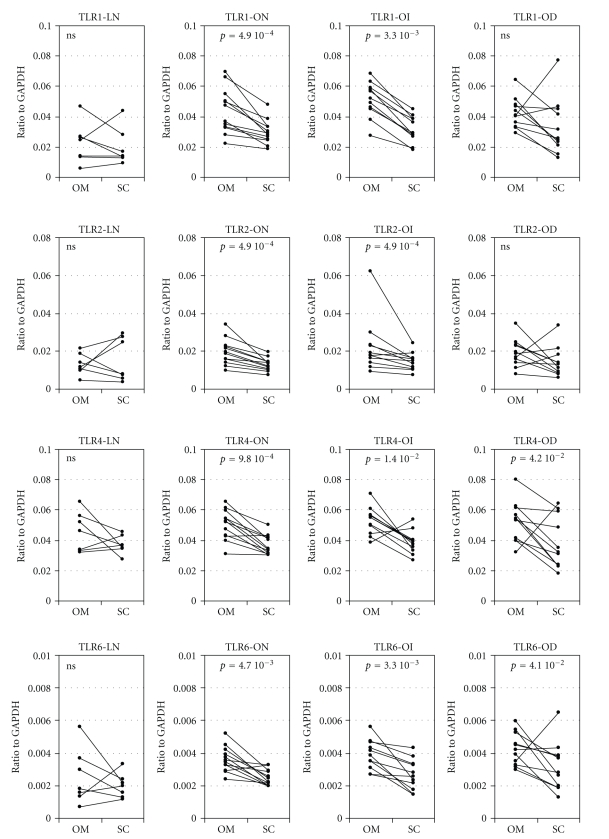
*TLR1*, *TLR2, TLR4*, and *TLR6* mRNA expression in adipose tissues according to localization (OM = omental, SC = subcutaneous;) and to phenotypic group (LN: lean normoglycemic; ON: obese normoglycemic; OI: obese glucose intolerant; OD: obese diabetic) in human subjects. The nonparametric paired Wilcoxon test was performed between subcutaneous and omental values and *P*-value is given in each corresponding panel (ns: *P* > .05).

We then analyzed independently each glucose tolerance subgroup (Figure 2). It showed a consistent higher expression of *TLRs* in omental adipose tissue. This difference was significant in obese normoglycemic and obese glucose intolerant groups, never in lean group and significant for *TLR4* and *TLR6* in diabetic subjects. We then performed Spearman's correlation analysis (nonparametric analysis based on rank) between each TLR expression and each depot (data not shown). For a given TLR, expressions was strongly correlated in omental and subcutaneous adipose tissues (e.g., individuals with high *TLR1* expression in the omental depot also showed a high subcutaneous *TLR1* level). In each given depot, a correlation between the four TLRs expression was also observed (e.g., individuals with a high *TLR1* expression in subcutaneous depot also display a high expression of all other TLRs in this depot). These results suggest a common mechanism of expression regulation.

Mann-Whitney analysis was performed to detect an association between TLRs expression and glycaemic status. No correlation between expression levels of *TLRs *and glycaemic status was observed among obese subjects. The only significant value obtained was between lean and obese normoglycemic subjects in omental tissue for *TLR1* and *TLR2* (*P* = 3.73 10^−3^ and 3.58 10^−2^, resp.)

### 3.2. Toll-Like Receptors Expression and *TLR2* Functionality in Murine 3T3-L1 Cell Line

**Figure 3:**
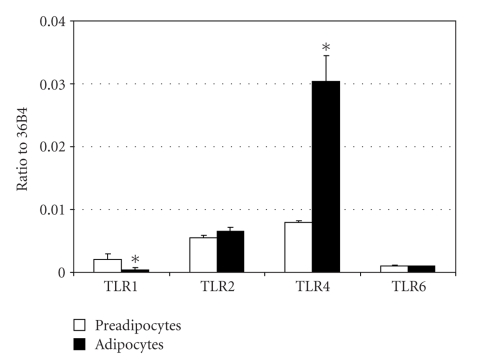
*TLR1*, *TLR2*, *TLR4*, and *TLR6* expression in 3T3-L1 cell line. Relative expression to *36B4* of genes coding for *TLR1* and *TLR2* was measured by qPCR in preadipocytes (white) or after ten-day differentiation of 3T3-L1 (black). Mann-Whitney analysis was performed between preadipocytes and differentiated adipocytes values (*, *P* < .05).

To determine whether toll-like receptors are expressed and functional in an adipocyte cell line model, total mRNA was isolated from 3T3-L1 preadipocytes as well as fully differentiated adipocytes, and qPCR was performed. Considering differentiated adipocytes, the expression of *TLR4* mRNA was 76 times higher than that of *TLR1, *4.6 times higher than that of *TLR2 *and 30 times higher than that of *TLR6*. This relative expression difference was also observed in preadipocyte but with a lower range (Figure 3). Differentiation into adipocyte has no significant impact on *TLR2* and *TLR6* expression levels while *TLR1* expression is lowered (5-fold) and *TLR4 *is enhanced (4-fold).

**Figure 4:**
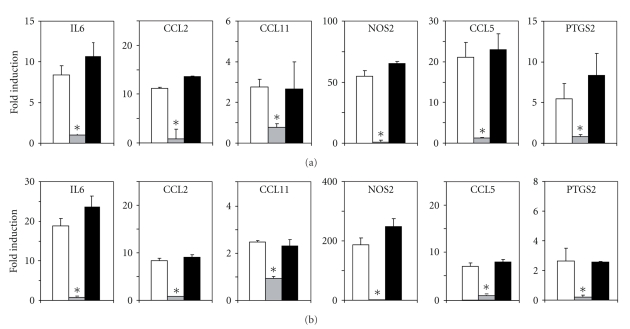
Induction of genes coding for *IL6, CCL2, CCL5, CCL11, NOS2*, and *PTGS2* was measured by qPCR in preadipocytes (a) or in differentiated 3T3L1 (b) after 4 hours stimulation with 1 ng/mL Pam3CSK4 in the absence (white) or presence of a blocking antibody (25 *μ*g/mL; grey) or an isotypic control (25 *μ*g/mL; black). Mann-Whitney analysis was performed between values obtained in absence or presence of antibodies (*, *P* < .05).

**Figure 5:**
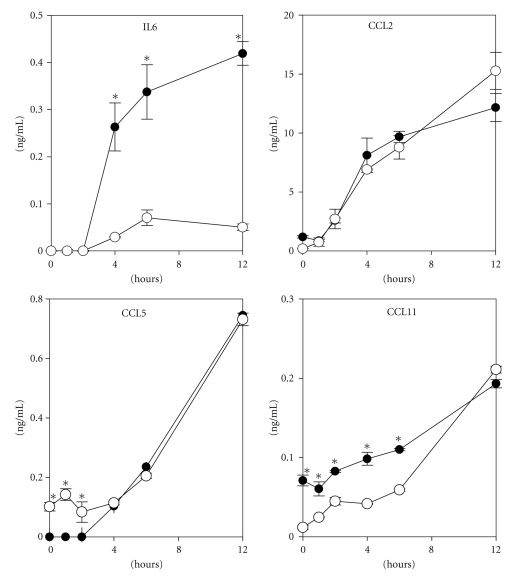
IL6, CCL2, CCL5, and CCL11 secretion was measured by ELISA in supernatants of preadipocytes (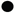) or differentiated 3T3-L1 (○) at different periods of time after stimulation with 1 ng/mL Pam3CSK4. All the results are presented as one experiment representative of at least three independent experiments.

We wanted to demonstrate that, besides *TLR4* as already published [15], other TLRs can be functional in 3T3-L1 cell line. We therefore stimulated 3T3-L1 preadipocytes and fully differentiated adipocytes with Pam3CSK4 (a *TLR1/TLR2* agonist) and measured the expression level of inflammation markers by qPCR after 4 hours of induction. mRNA coding for *IL6, CCL2, CCL11, NOS2, CCL5*, and *PTGS2* was highly induced by Pam3CSK4 stimulation in preadipocytes as well as in fully differentiated adipocytes (Figure 4). To further demonstrate the specificity of this stimulation and exclude the eventuality of *TLR4* stimulation via endotoxin contamination, we used a *TLR2* blocking antibody. Incubation with the *TLR2* antibody prevents the Pam3CSK4-induced expression of proinflammatory markers. A monoclonal antibody of the same isotype was used as a control and demonstrated no effect on the Pam3CSK4 induction. Therefore, the stimulation of proinflammatory markers by Pam3CSK4 can specifically be attributed to *TLR2* (Figure 5).

Cell supernatants were collected and cytokine and chemokine secretions were measured by ELISA at different time points after Pam3CSK4 stimulation. Protein secretion became detectable in supernatants four hours after stimulation. Preadipocytes were mainly responsible for release of IL6, whereas the CCL2, CCL5, and CCL11 concentrations observed were in the same range for both preadipocytes and adipocytes (Figure 5), demonstrating that adipocytes are able to secrete proinflammatory products via *TLR2/TLR1* activation in both states of differentiation. 

### 3.3. Effect of *TLR2/TLR1* Stimulation on 3T3L1 Differentiation

**Figure 6:**
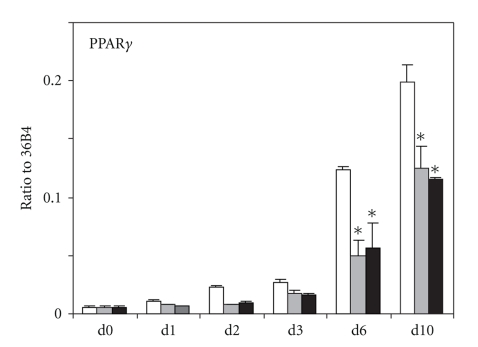
*P* *P* *A* *R* *γ* mRNA relative expression to *36B4* during 3T3-L1 differentiation (white) and during differentiation with continuous exposure to 1 ng/mL (grey) or 10 ng/mL (black) Pam3CSK4. Mann-Whitney analysis was performed between values obtained in absence or presence of Pam3SCK4 (*, *P* < .05).

We previously demonstrated that *TLR4* stimulation by LPS was able to impair adipocyte differentiation of 3T3-L1 cells [15]. To demonstrate that *TLR2/TLR1 *is also able to interfere with this process, we added 1 or 10 ng/mL Pam3CSK4 to the differentiating medium throughout the differentiation process. Microscopic observation of lipid-laden cells stained with oil-red-o showed a slight reduction of lipid droplets in 3T3-L1 cells cultured with Pam3CSK4 at day four. No significant differences were observed after 10 days (data not shown). Since *P* *P* *A* *R* *γ* is a key regulator of adipocyte differentiation [20], we monitored its expression level during the differentiation process. *P* *P* *A* *R* *γ* mRNA was detected 2 days after onset of differentiation and was further elevated at days 6 and 10. Treatment with Pam3CSK4 led to a 50% reduction of *P* *P* *A* *R* *γ* expression (Figure 6), demonstrating that *TLR2/TLR1* activation impairs adipogenesis.

## 4. Discussion

Obesity is defined as a low-grade chronic inflammatory disease associated with a moderate increase of circulating inflammatory factors [21]. This inflammation causes or worsens insulin resistance in insulin-responsive tissues such as adipose tissue, muscle, or liver. One of the causative factors of this inflammation process is the adipose tissue itself *via* its early infiltration with immune cells (mainly macrophages) [22] and *via* its autocrine and paracrine secretion of pro-and anti-inflammatory cytokines [23]. Importantly, it was shown that macrophage infiltration is more prominent in visceral fat than in subcutaneous fat [13, 24], thereby reinforcing the notion that intra-abdominal fat amount and metabolic abnormalities are correlated, as clearly shown by several epidemiological studies [10, 11]. 

One member of the TLR family, namely *TLR4*, was reported to participate to the development of inflammation and insulinresistance at the adipose level [5, 25]. We thus hypothesized that other functional members of the TLR family of innate immune receptors might also participate in these processes. Since, like *TLR4*, *TLR2* was demonstrated to sense fatty acids when dimerized either with *TLR1* or *TLR6* [6] we first focused our search on this receptor and on its dimerization partners *TLR1* and *TLR6.*

We found that *TLR4*, *TLR1*, *TLR2*, and *TLR6 *are significantly overexpressed in omental adipose tissue. Since a preferential macrophage infiltration into obese omental versus subcutaneous fat was demonstrated [26], we cannot exclude that toll-like receptors expression in adipose tissue is mainly due to macrophages as suggested by others [27], but has to be demonstrated since many other adipose tissue cell types can express toll-like receptors. We observed a strong correlation between each TLR expression within a given adipose depot and that at the individual level, each TLR expression is correlated positively in subcutaneous and omental depots. This suggests a common mechanism of regulation. In vitro, a cross-regulation between *TLR4* and *TLR2* expression was demonstrated after activation of 3T3-L1 cells with LPS, a *TLR4* agonist [28]. Additionally, LPS was also reported to slightly enhance *TLR1* expression in THP1 cells [29]. Whether LPS is the effector signal triggering overexpression of TLRs in human omental tissue could be hypothesized but other TLR ligands could also be involved. Endotoxemia was shown to participate in the initiation of obesity and insulin resistance [30], therefore serum LPS concentration as well as free fatty acid concentration would be interesting to be evaluated in relation to TLRs expression. Recently, resistin was demonstrated to be able to bind to *TLR4* [31], and other endogenous ligands such as HMGB1 or hyaluronan fragments were also reported as *TLR4* or *TLR2* activators [32]. Whether different TLR activators in obesity are of endogenous or exogenous origins or even of both, remained to be investigated and will be crucial for a better comprehension of this chronic inflammation.

We previously demonstrated that the inflammatory state associated with a decreased expression of lipogenic markers was more pronounced in diabetic subjects [14]. We could not reveal any differential expression according the prediabetic or diabetic status of the subjects. This might suggest that omental overexpression of toll-like receptors could play a role in early prediabetic phases of metabolic syndrome acquisition but not in further complications. 

Activation of *TLR2* by saturated fatty acids leads to the activation of MyD88-dependent signaling pathways whatever heterodimer is implied [6]. We here demonstrate that proinflammatory products can be synthesized by 3T3-L1 cells following *TLR2/TLR1* activation. We have used the well-known 3T3T-L1 murine preadipocyte cell line as an in vitro system for adipocyte generation to study the inflammatory response of both preadipocytes and adipocytes upon TLRs stimulation. The expression level of TLRs in 3T3-L1 cell line has not to be compared to what was obtained from human samples since adipose tissue is constituted of numerous different cell types (e.g., adipocytes, macrophages, and endothelial cells). Our purpose was to stress out the potential responsiveness of the adipocyte or of its precursors toward stimulatory compounds of the *TLR2* pathway. We had already shown that IL-6, certain chemokines (*CCL2*, *CCL2,* and *CCL11*), inducible nitric oxide synthase (*NOS2*), and cyclooxygenase-2 (*PTGS2*), all important mediators of inflammation, can be induced via *TLR4* activation in the 3T3-L1 cell line [15]. We demonstrate here that these molecules are also inducible specifically through *TLR2/TLR1* activation. *TLR4 *activation induced a lower secretion of *IL6*, *CCL5*, and *CCL11* in adipocytes when compared to preadipocytes, while *CCL2* secretion was similar in both differentiation states [15]. Following *TLR2* activation, only the secretion of IL-6 was lower in differentiated adipocytes than in preadipocytes. Similarly, we observed different induction factors for mRNA levels of the proinflammatory enzymes *NOS2* and *PTGS2* in this study of activation via *TLR2* and in the previous study of activation via *TLR4* [15]. Recently, an exhaustive study of *TLR1* to TLR9 activation of adipocyte with corresponding ligands was reported demonstrating a distinct response for each receptor [33]. As stated before, the pertinence of these observations will be strengthened when the “in vivo” genuine ligands will be described. We have observed that *TLR2 *and* TLR6* expression is constant throughout differentiation of 3T3-L1 cells, whereas *TLR1* and *TLR4* expression is modulated in fully differentiated adipocytes. Whether this could explain the weaker secretion of IL-6 observed in fully differentiated adipocyte can be hypothesized. We suggest that a fine regulation of the synthesis of proinflammatory mediators could be achieved in adipose tissue via differential expression and activation of TLR family members. As for *TLR4* stimulation, we were not able to detect any TNF*α* transcripts in Pam3CSK4-stimulated 3T3-L1 preadipocytes. This is in agreement with previous observation that showing TNF*α* release in human adipose tissue is mainly due to nonfat cells [34]. 

To conclude, we demonstrate here that *TLR2/TLR1* activation is able to interfere with adipocyte differentiation in the 3T3-L1 cell line, as previously described for *TLR4* [15]. This could occur either directly or via secretion of adipoctye-derived proinflammatory and antiadipogenic products. This observation is in agreement with our previous study demonstrating that the expression of lipogenic factors is reduced in omental adipose tissue in correlation with inflammation increase [14]. 

Beside their defense function of alerting the immune system of the presence of pathogenic microorganisms, TLRs can also sense dietary lipids. Therefore, it is tempting to speculate that detection of abnormal level or composition of these lipids will induce a physiological response. Our observations suggest that it might be the case and that, in addition to *TLR4*, other TLR family members, that are functional and present in adipocytes, could play this role. Unfortunately, the physiological response results in elicitation of a chronic omental adipose inflammation which contributes to metabolic syndrome.

## Figures and Tables

**Table 1:** General characteristics of the study subjects.

	Lean	Obese	Obese	Obese
		Normoglycemic	Glucose intolerant	Type 2 diabetic
	(*n* = 7)	(*n* = 12)	(*n* = 11)	(*n* = 11)
Age (yr)	42.6 ± 13.8	43.5 ± 5.0	40.7 ± 5.5	48.2 ± 7.1
BMI (kg/m2)	21.4 ± 2.4	44.2 ± 4.8	46.3 ± 4.8	48.8 ± 6.6
Fasting glucose (mmol/l)	4.38 ± 0.67	5.00 ± 0.50	5.48 ± 0.95	8.53 ± 2.13
Glucose 2h OGTT (mmol/l)	n.d.	6.18 ± 0.72	8.51 ± 1.13	n.d.
Insulin (mUI/l)	n.d.	11.23 ± 6.52	11.80 ± 3.82	19.92 ± 14.33

Mean value ± SD. Comparisons were made between the various categories of obese patients using the Kruskal-Wallis test; n.d. = not done.

## Abbreviations

CCL2: Chemokine (C-C motif) ligand 2 = MCP-1
CCL5: Chemokine (C-C motif) ligand 5 = RANTES
CCL11: Chemokine (C-C motif) ligand 11 = eotaxin
GAPDH: Glyceraldehyde-3-phosphate dehydrogenase
IL6: Interleukin 6
NOS2: Nitric oxide synthase 2A = iNOS
OGTT: Oral Glucose Tolerance test
Pam3CSK4: N-Palmitoyl-S-[2,3-bis(palmitoyloxy)-(2RS)-propyl]-[R]-cysteinyl-[S]-seryl-[S]-lysyl-[S]-lysyl-[S]-lysyl-[S]-lysine
PTGS2: Prostaglandin-endoperoxide synthase 2 = cyclooxygenase 2 = COX2
36B4: Acidic ribosomal phosphoprotein.
